# Information needs of Asian and White British cancer patients and their families in Leicestershire: a cross-sectional survey

**DOI:** 10.1038/sj.bjc.6601774

**Published:** 2004-04-06

**Authors:** D Muthu Kumar, R P Symonds, S Sundar, K Ibrahim, B S P Savelyich, E Miller

**Affiliations:** 1 Department of Oncology, Leicester Royal Infirmary, University of Leicester, Leicester, LE1 5WW, UK; 2 University of Nottingham, Nottingham NG7 2RD, UK; 3 Department of Clinical Psychology, University of Leicester, Leicester LE1 7RH, UK

**Keywords:** information needs, family and patient preferences, Asian patients

## Abstract

The aim of this questionnaire survey was to find the information needs of British Asian cancer patients. An additional objective was to find the extent of family involvement when the patient was given the cancer diagnosis and the patients' views about information disclosure. We interviewed 82 Asian patients and 220 random white control patients. More white British patients gave positive answers to the statement ‘I want as much information as possible’ than Asian patients (93.1 *vs* 77.5%, *P*⩽0.001). However, 92.6% of Asian patients wanted to know if they had cancer. Many more Asians (66.2 *vs* 5.1%, *P*<0.001) indicated the general practitioner (GP) as the preferred source of information. This may be because 56% of English-speaking Asian patients would prefer to discuss their illness in their mother tongue. In Leicester, many Asian patients have Asian GPs. The vast majority of both Asian and British patients agreed that family or friends should be present when patients are given the cancer diagnosis. However, Asians were more likely to be alone (24 *vs* 15%, *P=*0.008) when told they have had cancer. The majority of patients (both white British and Asian) want to control the disclosure of information to relatives and friends and would like to be present at doctor/family meetings.

Patients cope better with cancer when provided with clear adequate information tailored to their needs (Fallowfield *et al*, 1990). A 250 patient stratified sample typical of West of Scotland cancer patients (Meredith *et al*, 1996) and a more heterogeneous study of 2331 patients (Jenkins *et al*, 2001) showed the vast majority of British cancer patients want to know the diagnosis and specific details about treatment and prognosis. However, the information needs of British non-white ethnic minorities have never been studied.

Leicester is a multicultural city of 280 000. The 2001 Census (Office for National Statistics, 2001) described only 63.9% of the population as white. The biggest minority population (25.7%) was described in the census as Indian, although some of these people would use a different name such as African/Asian to describe their origin. It has been predicted that by 2011, 50% of the population of Leicester will be of Asian origin (Rex, 1999).

Cancer registration statistics collected between the start of 1990 and the end of 1999 for the city of Leicester showed that South Asian patients had a lower cancer incidence than non-South Asians (Smith *et al*, 2003a). When adjusted for age and socioeconomic deprivation, incidence rate ratio (IRR) was 0.61 for male and 0.75 for female population. This pattern changed significantly with age. Although older Asians had much lower rates of cancer than the rest of the population, younger Asians were at increased risk compared with non-Asians. Comparing incidence rate ratios between 1990–1994 and 1995–1999 there has been a marked rise in the incidence of colorectal cancer (IRR=2.33) and breast cancer (IRR=1.37) in Asian women. In contrast, these rates decreased in the rest of the population. There has also been a rise in the incidence of prostate cancer (IRR=1.71) and lung cancer (IRR=1.39) in Asian men. The trend for more lung cancer is likely to continue owing to higher smoking rates in Asian men aged 30–49 years than those aged 50–74 years (Smith *et al*, 2003b).

The aim of the study was to evaluate the information needs of Asian patients and depth of information required. This was compared to the information needs of white British patients. An additional objective was to find the extent of family involvement during the consultation when the patient was given the diagnosis and the patients' views about disclosing information to family or close friends.

Although most (85%) of our Asian patients speak English, the majority consider an Asian language, particularly Gujarati, as their mother tongue. Currently, we have a 23-min video available in Gujarati and Hindi. This gives basic information about cancer and the main treatments. However, we have no written information about radiotherapy, chemotherapy and the treatment of tumours at specific sites available in Asian languages. We wanted to find out if our Asian patients prefer to discuss their condition and treatment in their mother tongue and if there is a need for written information in Asian languages.

## PATIENTS AND METHODS

This study was approved by the Leicestershire Ethics Committee.

Between September 2001 and September 2002, as many Asian patients as possible attending the Oncology Department of Leicester Royal Infirmary were invited to take part in this study. They were interviewed by a doctor or radiographer who could speak English, Gujarati, Hindi, Punjabi, Urdu or Tamil during a course of radiotherapy or chemotherapy. Eighty-two Asian patients completed the study, 15 declined to be interviewed. Ten questionnaires were posted to patients who attended the Oncology Department once or twice only and none were returned.

In total, 107 out of 138 Asian (77.5%) patients registered during this period were invited to take part in this study.

The majority of Asian patients were interviewed in English (73.2%), the rest in Gujarati (20.7%) or Hindi (2.4%). The 220 white British patients who agreed to take part in the study were selected randomly. Thirty patients (20%) declined to participate. Survey patient characteristics are listed in Table 1

**Table 1:** Demographic data of study patients

	**Asian**	**White British**
Total	82	220
Characteristic		
Male^a^	35 (42.7)	75 (34.2)
Female^a^	47 (57.3)	144 (65.8)
Median age (years)^b^	50	62
Age range (years)	18–77	31–89
Ethnic origin		
Indian	56 (68.3)	
British Asian	18 (22.0)	
British	1 (1.2)	214 (97.3)
Pakistani/Bangladeshi	6 (7.4)	
Continent/country of birth		
Africa	37 (45.1)	
Indian	33 (40.2)	
Britain	6 (7.3)	213 (96.8)
English speakers	69 (84.1)	220 (100)
Non-English speakers	13 (15.9)	
Other languages		
Gujarati	60 (73.2)	
Hindi	39 (47.6)	
Punjabi	21 (25.6)	
Urdu	17 (20.7)	

Values are numbers (percentage in parentheses). Figures less than 100% reflect missing or ‘other’ category data.

a *P* 0.18, Fisher's exact test (two-sided).

b *P*<0.001, unpaired *t*-test.

. Some Asian patients spoke two or more languages other than English.

The most common tumour site and type of treatment are given in Table 2

**Table 2:** Most common tumour site and treatment modality

	**Asian**	**White British**	***P*-value**
			
Tumour site			
Breast	27 (32.9)	90 (40.9)	
Colon/rectum	10 (12.2)	39 (17.7)	
Prostate	10 (12.2)	35 (15.9)	
Head and neck	7 (8.5)	3 (1.4)	
Other	28 (34.2)	53 (24)	
Treatment intent^a^			
Potentially curative	72 (88.9)	173 (80.8)	0.19^b^
Palliative	9 (11.1)	41 (19.2)	
Treatment type^c^			
Surgery	53 (67.9)	152 (69.7)	0.78^a^
Radiotherapy	69 (88.5)	171 (79.2)	0.09^a^
Chemotherapy	43 (53.8)	104 (48.4)	0.43^a^

Values are numbers (percentage in parentheses).

a Excluding seven patients with uncertain intent.

b Fisher's exact test (two-sided).

c Patients may have received more than one treatment modality.

. Many patients had more than one treatment modality. Patients' views about information were elicited by a questionnaire widely used in the past (Cassileth *et al*, 1980; Jenkins *et al*, 2001) and modified in the light of experience in the west of Scotland (Meredith *et al*, 1996). It consisted of two parts, one of which described their general preference for information. Six additional questions were asked about information needs relating to their illness. We used balanced five/three-point-Likert-like scales to record patients' information needs—(a) Strongly agree – strongly disagree, (b) absolutely need information – definitely do not want this information, (c) want as much information as possible – do not want to know details.

Patients were asked the preferred source of information (GP, hospital specialist or nurse). Asian patients were asked about the importance of leaflets in Asian languages and the opportunity of discussing their illness in their mother tongue. The place where diagnosis of cancer was given was ascertained and whether they were accompanied by family members or a close friend and whether permission was sought by the doctor to discuss the patient's diagnosis in the presence of friends or relatives was noted. Additionally, patients were asked if everyone they wanted was present, if they knew if family or friends were welcome at the consultation and how important was the presence of family members or friends at this consultation. The importance of permission being granted prior to any disclosure to relatives and whether the patients wanted to be at this meeting was assessed. The results were tabulated in an Access database. All analyses were performed using the SPSS/PC (version 11.0) statistical program. (SPSS Inc., Chicago, IL, USA).

Statistical significance was assessed by the Mann–Whitney test, *χ*^2^ test, Fisher's exact test and unpaired *t*-test. In particular, the responses to questionnaires on the Likert scale were treated as ordered categories and were analysed by the Mann–Whitney test (Altman, 1991). *χ*^2^ test was not appropriate for these data sets since more than 20% of cells had an expected frequency of less than 5 and collapsing the categories would have led to loss of valuable information.

## RESULTS

The majority of Asian patients were born outside the UK (92%). Only six were born in Britain. Asian patients were significantly younger (*P*=0.001) (median age 50 years) compared to white patients (median age 62 years) reflecting the lower average age of the ethnic minority nationwide. There were more female patients in both arms of the study as more than a third of all patients were suffering from breast cancer. Slightly more Asian patients (88.9 *vs* 80.8%) received potentially curative treatment (*P*=0.19). There was no difference in usage of different treatment modalities (surgery, radiotherapy or chemotherapy) between the two groups (Table 2).

### Information needs questionnaire

More white British patients wanted as much information as possible (93.1 *vs* 77.5%) and this difference was statistically significant (*P*⩽0.001) (Table 3

**Table 3:** Attitude to information

	**Asian**	**White British**
Want as much information as possible	62 (77.5)	202 (93.1)
Want additional information only if it is good news	10 (12.5)	5 (2.3)
Do not want to know details	8 (10.0)	10 (4.6)

Values are numbers (percentage in parentheses). *P*<0.001 (Mann–Whitney *U*-test (two-tailed-test).

). However, although only 77.5% of Asian patients wanted as much information as possible, 92.6% said they absolutely needed to know or would like to know if they had cancer. Similarly, a high percentage of Asian patients wanted to know the specific name of the illness (93.8%), week-by-week progress (86.4%), the chance of cure (84.0%), all possible treatment (87.7%) and all possible side effects (85.2%). Similar answers were given by white British patients, indicating a similar high demand for this information (Table 4

**Table 4:** Responses of 82 asian and 220 white british cancer patients to specific questions about need for information^a^

	**Absolutely need information**		**Would like this information**		**Have no views**		**Would not like this information**		**Definitely do not want this information**		
**Question**	**Asians**	**White British**	**Asians**	**White British**	**Asians**	**White British**	**Asians**	**White British**	**Asians**	**White British**	***P*-value^b^**
Specific name of illness	47 (58.0)	76 (34.7)	29 (35.8)	132 (60.3)	2 (2.5)	8 (3.7)	0	3 (1.4)	3 (3.7)	0	0.001
Whether illness is cancer	57 (70.4)	145 (65.9)	18 (22.2)	72 (32.7)	1 (1.2)	1 (0.5)	0	2 (0.9)	5 (6.2)	0	0.71
What is week-by-week progress	39 (48.1)	85 (39.4)	31 (38.3)	102 (47.2)	3 (3.7)	20 (9.3)	3 (3.7)	7 (3.2)	5 (6.2)	2 (0.9)	0.34
What is chance of cure	52 (64.2)	112 (50.9)	16 (19.8)	97 (44.1)	8 (9.9)	4 (1.8)	2 (2.5)	6 (2.7)	3 (3.7)	1 (0.5)	0.27
What are all possible treatments	48 (59.3)	112 (50.9)	23 (28.4)	101 (45.9)	3 (3.7)	6 (2.7)	4 (4.9)	1 (0.5)	3 (3.7)	0	0.61
What are all possible side effects of treatment	50 (61.7)	106 (48.2)	19 (23.5)	102 (46.4)	6 (7.4)	9 (4.1)	2 (2.5)	3 (1.4)	4 (4.9)	0	0.25

Values are numbers (percentage in parentheses).

a Crosstabulation excludes missing values.

b Mann–Whitney *U*-test.

).

Two important subgroups wanted less information. Only 65.5% of Asians older than 60 years wanted as much information as possible compared to 91.3% of white British older than 60 years (*P*<0.001). However, 86.2% of Asians aged 60 years or older wanted to know if they had cancer. Four out of 12 non-English speakers (33%) wanted no details of their illness and only five out of 12 (41.7%) wanted as much information as possible. Even so, eight out of 12 (67%) wanted to know if they had cancer.

### Preferred source of information

**Figure 1:**
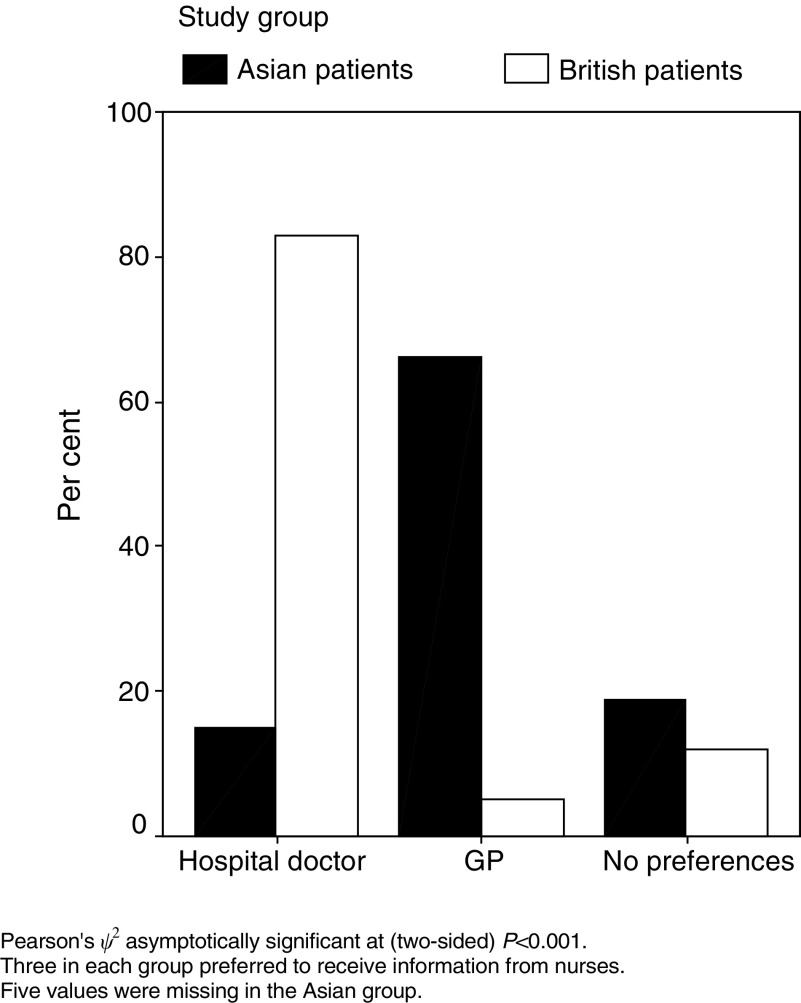
Preferred giver of diagnosis.

The majority of white British patients (82.9%). wanted to be given the diagnosis and other information by a hospital specialist (see Figure 1

). The views of the majority of Asian patients were significantly different (*P*<0.001). A total of 66.2% of Asian patients named their general practitioner (GP) as the preferred source of information compared to only 5.1% of white British patients. Only three patients in each group chose a nurse as their ideal information source.

### Preferred language for information and discussion

Thirty-three (56%) of English-speaking Asian patients had an absolute need or would like to discuss their illness in their own language. A similar number of English speakers (32–53%) would like to read leaflets in their own language. Not unexpectedly, 82.4% of Gujarati speakers who did not speak English had an absolute need or would like to discuss their illness in Gujarati and 95% wanted leaflets in that language.

### Family involvement

Most patients were given the diagnosis of cancer in an outpatient clinic. There was a statistically significant trend for more Asian patients to be alone at this critical consultation (24 *vs* 15%, *P*=0.008). Slightly more Asian patients were more likely to be accompanied by family members than were white British patients (71.3 *vs* 66.7%), but on the other hand, more white patients came with friends (5.0 *vs* 18.3%).

The vast majority of both Asians (88.9%) and white British patients (92.2%) agreed or strongly agreed with the view that family or friends should be present when the patient is given the diagnosis of cancer. A statistically significant proportion (26%) of Asian patients were not aware that friends or family members could come to the consultation (Fisher's exact test: *P*=0.04). However, the majority of patients want to control the disclosure of information to relatives and friends. In all, 70.0% of Asian and 69.8% of white British patients felt that it was important that the doctor asked their permission before speaking to others. Moreover, 65% of Asians and 71.1% of white patients would have liked to be present when others were informed.

The only statistically significant subgroup (*P*⩽0.01) who did not want to be present at doctor family discussions were Asians older than 60 years with only 39.3% wanting to attend (Tables 5

**Table 5:** People present and place of diagnostic interview^a^

	**Asian**	**White British**	***P*-value**
Place of diagnosis			
Outpatients	62 (76.5)	182 (83.4)	0.35^b^
Ward	15 (18.5)	30 (13.8)	
Elsewhere	4 (4.9)	6 (2.8)	
When given the diagnosis who was present			
Friend	4 (5.0)	40 (18.3)	0.008^b^
Family	57 (71.3)	146 (66.7)	
Alone	19 (23.8)	33 (15.1)	
Aware friends/family were welcome at the consultation			
Yes	59 (73.8)	184 (84.4)	0.04^c^
No	21 (26.3)	34 (15.6)	
Was everyone you wanted present at the time			
Yes	56 (70.0)	187 (85.8)	0.004^c^
No	24 (30.0)	31 (14.2)	

Values are number (percentage in parentheses).

a Crosstabulation excludes missing values.

b Pearson's *χ*^2^ test (two- sided).

c Fisher's exact test (two-sided).

and 6

**Table 6:** Views of Asian and white British on information giving to family and friends^a^

	**Strongly agree**		**Agree**		**Not bothered**		**Disagree**		**Strongly disagree**		
	**Asians**	**White British**	**Asians**	**White British**	**Asians**	**White British**	**Asians**	**White British**	**Asians**	**White British**	***P*-value^b^**
Important diagnosis is given when family or friend is present	50 (61.7)	78 (35.6)	22 (27.2)	124 (56.6)	5 (6.2)	15 (6.8)	1 (1.2)	2 (0.9)	3 (3.7)	0	0.001
Important doctor asks my permission before giving information to family	32 (40.0)	54 (25.1)	24 (30.0)	96 (44.7)	17 (21.3)	60 (29.7)	0	5 (2.3)	7 (8.8)	0	0.24
Important to be present when doctor tells the family about my illness	26 (32.5)	68 (31.6)	26 (32.5)	85 (39.5)	19 (23.8)	54 (25.1)	4 (5.0)	8 (3.7)	5 (6.3)	0	0.42

Values are numbers (percentage in parentheses).

a Crosstabulation excludes missing values.

b Mann–Whitney *U*-test.

).

## DISCUSSION

There are a number of anecdotal opinions about Asian patients that have never been substantiated by research. It is said that the diagnosis of cancer is culturally unacceptable and Asians adopt a more passive role leaving decision-making to the doctor or family. This study does not support these assertions.

In common with white Leicestershire patients, the majority of Asian patients wanted full information about their illness. In particular, 92.6% wanted to know if they had cancer. A small minority wanted very little information, particularly patients older than 60 years and non-English speakers. In clinical practice, this important subgroup should be identified and their views respected. In Leicester, many Asian patients consult Asian GPs. It is noteworthy that 56% of English-speaking Asian patients want to discuss their illness in their mother tongue. The cultural and linguistic bonds between these doctors and patients may explain why most (66.2%) Asian patients prefer to be given the cancer diagnosis by their GP. In practice, most patients will be given the diagnosis by a hospital specialist. However, patients, if they wish to, should be encouraged to discuss their illness further with their GP, perhaps in an Asian language. To facilitate these discussions, a letter should be rapidly sent to the GP, listing treatment alternatives, recommendations for treatment and an estimate of prognosis. Although 53% of English-speaking Asian patients would like to read leaflets in an Asian language, we feel that written information is only a partial substitute for adequate discussion with a medical person who speaks their mother tongue.

There is evidence that women from ethnic minority groups do not come forward for breast and cervical screening. Uptake can be improved by health promotion programmes coordinated by bilingual health workers of the same ethnic minority (Editorial, 2001). However, our study indicates a definite preference among Asian patients for the GP to give the critical information about diagnosis, prognosis and treatment options and not a nurse or even a hospital specialist. Two studies in ethnic minority patients in the United States (Guidry *et al*, 1998; O'Malley *et al*, 1999) have shown that doctors are the most important sources of health information for some minority groups, particularly American blacks. Currently, both consultant and secretarial time is in short supply. In order to implement our suggestion that the GP is rapidly sent a comprehensive letter to facilitate discussion with Asian patients, additional resources would have to be provided.

The majority of patients were told that they had cancer in outpatients. This reflects changes in modern hospital practice. We were surprised to find that more Asian patients were alone (23.8%) than white patients (15.1%) at the critical consultation. In all, 30% of Asians and 14.2% of white patients said that not everyone they wanted was present at the consultation. Although 73.8% of Asian and 84.4% of white patients were aware that relative or friends were welcome at the consultation, we feel that when the patient is to be given bad news, the appointment letter should also include a formal invitation to bring a relative or friend.

The results of this study strongly support the practice of asking the patient's permission before disclosure of confidential information. More than two-thirds of the patients in this study wanted to be present at this meeting between the doctor and family members. These results are very similar to a 30-patient retrospective study in general practice of patients treated for cancer 1–7 years ago. Only six favoured unconditional disclosure of information to their family (Benson and Britten, 1996). We do not know of any other survey of information needs of Asian or any other ethnic minority group of patients with cancer in Britain. Indeed, we do not know of any similar study of cancer patients in the Indian subcontinent. A survey of 148 patients in the Bangalore district who had recovered following hospitalisation for a wide variety of health problems found that 94% wanted to know the nature of their illness and 90% the cause. Interestingly, 30% felt that insufficient information was provided about the nature of their illness (Sriram *et al*, 1991).

This study reinforces data from the west of Scotland (Meredith *et al*, 1996) and the large survey of Jenkins *et al* (2001), which show that most cancer patients want as much information as possible. This is also true of Asian patients in Leicestershire.
